# Adapting World Health Organization Disability Assessment Schedule 2.0 for Nepal

**DOI:** 10.1186/s40359-021-00550-5

**Published:** 2021-03-17

**Authors:** Ajay Risal, Dipak Kunwar, Eliza Karki, Shambhu Prasad Adhikari, Inosha Bimali, Barsha Shrestha, Subekshya Khadka, Are Holen

**Affiliations:** 1 Dhulikhel Hospital, Kathmandu University Hospital, Dhulikhel, Kavre Nepal; 2 Department of Psychiatry, Dhulikhel Hospital,, Kathmandu University School of Medical Sciences,, Kathmandu University Hospital, GPO Box 11008, Dhulikhel, Kavre, Nepal; 3 Department of Physiotherapy, Dhulikhel Hospital, Kathmandu University School of Medical Sciences, Dhulikhel, Kavre, Nepal; 4 Department of Mental Health, Norwegian University of Science and Technology, Trondheim, Norway

**Keywords:** Cultural adaptation, Disability assessment, Epidemiological research, Nepali version, WHODAS

## Abstract

**Background:**

Disability is a vital public health issue for health care programs. Affluent countries usually prioritize disability-related research, while often it remains neglected in resource-poor countries like Nepal. The aim of this study was to make available a translated and culturally adapted version of the World Health Organization Disability Assessment Schedule 2.0 (WHODAS 2.0) for measuring disability in the Nepalese population.

**Methods:**

WHODAS 2.0 (12-items version) was translated into Nepali using a standard forward–backward translation protocol. Purposive and convenience recruitment of participants with psychiatric disabilities was done at the Psychiatry services in a tertiary care hospital. Age and gender-matched participants with physical disabilities were selected from the Internal Medicine department, and participants with no disability were recruited from their accompanying persons. A structured interview in Nepali including the translated WHODAS 2.0 was administered to all participants. Exploratory factor analysis and parallel analysis assessed the construct validity. Content validity was explored, and a quality of life instrument was used for establishing criterion validity. Reliability was measured via Cronbach alpha. Mann–Whitney test explored score differences between the disabled and non-disabled.

**Results:**

In total, 149 persons [mean age: 40.6 (12.8); 43.6% males, 56.4% females; 61.7% disabled, 38.3% non-disabled] consented to participate. Parallel analysis indicated that a single factor was adequate for the Nepali WHODAS version that captured 45.4% of the total variance. The translated scale got a good Cronbach alpha (= 0.89). Satisfactory construct, content and criterion validity was found. The WHODAS total scores showed a significant difference between the disabled and non-disabled (U = 2002.5; p = 0.015). However, the difference between psychiatric and physical disabilities was not significant, which underscores that the scale is rating disability in general.

**Conclusion:**

The one-factor structure of the translated and culturally adapted Nepali-version of WHODAS 2.0 showed acceptable validity and an adequate reliability. For epidemiological research purposes, this version of WHODAS 2.0 is now available for measuring global disability in Nepal.

## Background

Advances in health care have increased longevity, reduced morbidity and mortality. The functional ability of disabled individuals to participate in the daily routines at home, at work, and in society have come more into focus [1]. Limited functional ability is a significant clinical condition and a major public health issue [2]; its assessment is vital for priority-setting and performance of health efforts in any country.

In the bio-psycho-social health model, disability should be regarded as a multidimensional construct that includes interactions between individuals, their physical and social attributes [1]. The disability concept should be neutral with respect to the etiology, pathology or other characteristics [3]. Widespread cultural diversity and distinct socio-behavioral characteristics of a population impose unique challenges in disability assessments [4, 5].The International Classification of Functioning, Disability and Health (ICF) has suggested an operational definition of ‘disability’ as a decrement in domains of functioning at the body, person or societal levels; the concept is considered to be an umbrella covering any kinds of impairment, activity limitations, and restrictions in participation [3, 6].

At the outset, the ICF model was criticized for being impractical in routine clinical settings [1]. In response, the World Health Organization (WHO) developed the WHO Disability Assessment Schedule (WHODAS) to provide a standardized way of rating disability across conditions and cultures [1, 6]. The original WHODAS, published by WHO in 1988, was only concerned with psychiatric in-patients [7]. WHODAS 2.0, however, an altogether different instrument, was developed to address core components and constructs of the broad ICF definition [1, 8] for any disability. The instrument gives a general assessment of disability, and it has been used for multiple purposes and in different settings, such as in population surveys, patient related outcome measures, and clinical trials [1, 6, 8]. It has also been extensively used in cross-cultural research of general populations, but also among those with physical conditions in addition to those with mental, neurological, and substance use disorders [8].

Studies from different parts of the world and diverse socio-cultural settings tend to retain the original one-factor structure of the WHODAS 2.0 instrument [1, 8]. A validity survey among persons with various mental and physical disorders in Australia revealed a two-factor structure [9], while a recent online validity study of persons with anxiety and stress disorders in Sweden resulted in three factors [10]. However, both these studies later came up with a strong latent variable, the ‘global disability’ illustrating a viable one-factor solution. A cross-cultural adaptation of the Hebrew version [4] as well as the Chinese version [11] demonstrated a stable one-factor structure. The unidimensionality of the instrument has also been identified in several cross-cultural studies [3, 12, 13].

Psychometrically, the one-dimensional structure of the WHODAS 2.0 has generally demonstrated acceptable reliability and validity [1, 8]. It has been used in almost 100 countries and translated into around 50 languages and dialects, and applied in 27 research settings [3].

WHODAS 2.0 has been applied in the World Mental Health (WMH) surveys [14] as well as in many national surveys: Australia [9], New Zealand [15], Europe [16, 17], Ireland [18], Sweden [10], France [19], and in different low-and-middle-income (LAMI) countries [13, 20]. Most of the validation studies were done in relation to psychiatric populations. Some were conducted among persons with physical health conditions like chronic illnesses such as visceral disability [11], pulmonary hypertension [21], hand injuries [4], fall-related injuries [22], low back pain [5, 23], Huntington’s disease (HD) [24]; and among patients with comorbid psychiatric disorders with a variety of medical conditions [14].

In Nepal, epidemiological and clinical research on disability still remains neglected [25, 26]; one reason is the absence of a culturally adapted and valid instrument. Although a couple of prior studies in Nepal used WHODAS 2.0 [27, 28], the instrument has not been validated, so far.

With this backdrop, we aimed to develop a translated and culturally adapted version that can demonstrate adequate psychometric properties of the WHODAS 2.0, and in this way, we wanted to contribute to the pool of psychometrically sound instruments for epidemiological and clinical use in Nepal.

## Methods

### Ethics

This study was part of a larger research project addressing disability and quality of life (QoL) of persons with mental illness reporting to a tertiary care University Hospital in the Kavre District, Nepal. The Institutional Review Committee of Kathmandu University School of Medical Sciences (IRC-KUSMS), Dhulikhel Hospital (DH), Kavre, Nepal, approved of the study protocol (approval number 02/20). Informed consent was obtained from all invited participants confirmed either by signature or fingerprint according to their literacy status.

### Design and sampling procedure

This was a cross-sectional study. Purposive and convenience recruiting of participants was done among consecutive patients and their accompanying persons when visiting the Psychiatry or the Medical Out-patients Department (OPD) of DH if they met the inclusion criteria stated below. In line with the definition of ICF, those who had any current psychiatric disorder as per the International Classification of Diseases (ICD-10) criteria [29] that had lasted at least one year were enrolled as having a psychiatric disability. Patients with chronic medical illnesses from the Internal Medicine OPD were enrolled as having a physical disability. Age (± 5 years) and sex-matched accompanying persons served as controls if they had no history of mental disorder and no chronic and debilitating medical disease. In addition, they should have no family history of mental illnesses. Accordingly, the following three groups of participants were parts of the study: Patients with a psychiatric disability, patients with physical disability, and finally, participants without any disability.

In the subsequent comparisons, participants with a psychiatric disability and/or a physical disability were merged into to one large group that represented the disabled in general.

The inclusion criteria were an age-range within 18–65 years and no cognitive impairment. Every participant gave their consent to take part in the study. The interview was made in the Nepali, which is the *lingua franca* of the country, and all participants understood it well [30].

### Study variables

A face-to-face structured interview was carried out by researchers from the Psychiatry department (AR, DK, EK, BS, and SK). The interview included questions and issues regarding:

1. Personal and demographic characteristics: age, sex (male or female), residence (urban or rural), literacy status (literate or illiterate; based on the ability to read and write), marital status (married vs. unmarried; no one were divorced or widowed in this population), family size, family type in household (nuclear or extended), and occupational status (employed, housewife, unemployed)
2. Socio-economic status (SES) assessed according to **“**Kuppuswamy’s Socio-economic Scale adjusted for Nepal” [31]
3. Family History of Psychiatric Illness (yes or no)
4. Disability related factors: Disabled (either psychiatric and/or chronic physical illnesses) or non-disabled (absence of any disability).

## Study instruments

### Kuppuswamy’s socio-economic scale adjusted for Nepal

Assessment of the financial welfare of a person is difficult in an agricultural country like Nepal; abundance of self-produced items makes monetary income less important [30]. Kuppuswamy’s socioeconomic scale, originally developed in India [32], seems useful to overcome this obstacle. For a rough estimate of the SES of the family that the patient belongs to, a combined score of educational and occupational status of the head of the family, and the total monthly family income is calculated. The scale has been revised from time to time. Recently, it has been modified and standardized for Nepal in relation to the current economic status of the country [31]. For this study, we used the most recent version. The potential total score ranges from 3–29. Based on the total score, the SES of a family was categorized into: Upper (above 25), Middle (11–25), and Lower (10 and below) [31].

### World Health Organization Disability Assessment Schedule 2.0

The World Health Organization Disability Assessment Schedule 2.0 (WHODAS 2.0) is an attempt to obtain a graded disability assessment of a person. Mainly, the scale is based on activity limitations and participation restrictions [1]. Conceptually, it includes six different adult life domains: cognition (understanding and communication); mobility (getting around); self-care; getting along with people (interpersonal relationships); life activities (work and household roles); and social participation. Impairment in any of these life domains is rated as a degree of ‘disability’ [9]. The questions have the timeframe of the last 30 days. The scale has two versions: 12-items and 36-items; both have been successfully used for research purposes [3]. In the original English format, the 12-items version explained 81% of the variance in the 36-items scale [8]. As illustrated in the Table 1A, pairs of two items correspond to each of the six domains of the original WHODAS [3].

**Table 1:** WHODAS 2.0 12-items version & WHOQoL-8 Instruments: Items and domains

A. WHODAS 2.0*	
WHODAS Items	WHODAS domains
In the last 30 days, how much difficulty did you have in:	
S3. Learning a new task, for example, learning how to get to a new place?	Cognition (Understanding and Communication)
S6. Concentrating on doing something for ten minutes?	
S1. Standing for long periods such as 30 min?	Getting around (Mobility)
S7. Walking a long distance such as a kilometer [or equivalent]?	
S8. Washing your whole body?	Self-care
S9. Getting dressed?	
S10. Dealing with people you do not know?	Getting along with people
S11. Maintaining a friendship?	
S2. Taking care of your household responsibilities?	Life activities (Activities at home, work, and school)
S12. Your day-to-day work/school?	
S4. How much of a problem did you have in joining in community activities (for example, festivities, religious or other activities) in the same way as anyone else can?	Social participation
S5. How much have you been emotionally affected by your health problems?	

B. WHOQoL-8	
Items in the WHOQoL-8 scale	QoL domains
1. How would you rate your quality of life?	Global
2. How satisfied are you with your health?	
3. Do you have enough energy for everyday life?	Physical
4. How satisfied are you with your ability to perform your daily living activities?	
5. How satisfied are you with yourself?	Psychosocial
6. How satisfied are you with your personal relationships?	
7. Have you enough money to meet your needs?	Environmental
8. How satisfied are you with the conditions of your living place?	

^*^Reproduced with permission from WHO [39]

Being a short, and easily administrable in low-resource settings like Nepal, we decided to use the 12-items version. Each of the twelve items is rated from 0 (no disability) to 4 (complete disability). The potential total global disability score can range from 0 to 48.

The 12 items of the WHODAS 2.0 has strong correlations with one latent ‘general disability’ factor that covers tasks related to six central life-domains [8]. According to international reviews, the 12-item scale has acceptable psychometric properties with an overall reliability (alpha = 0.98) and an item-total correlation in the range of 0.59–0.94 [1, 8]. WHODAS 2.0 has been found suitable for clinical as well as epidemiological population studies when using the self-administration or interview approach [9].

### World health organization quality-of-life 8-question scale (WHOQoL-8)

The WHOQoL-8 is a short, efficient, and useful quality of life assessment scale [33]. Conceptually, eight items cover the four domains of the original 26—items brief version WHOQOL [33, 34]: global, physical, psychosocial, and environmental. As shown in the Table 1B, pairs of two items correspond to each of the four QOL domains. WHOQOL-8 has demonstrated acceptable psychometric properties in different studies and sociocultural settings [35]. The scale has been translated and culturally adapted for use among the Nepali population with an acceptable internal consistency (Cronbach’s alpha 0.74) [36, 37]. Each item of the WHOQoL-8 is rated on a five-point scale, scored from 1 (worst) to 5 (best); the sum score has a potential range from 5 to 40. Higher sum scores indicate better QoL. In this study, the instrument was used for assessing the criterion validity of the WHODAS 2.0.

### Cross-cultural translation and adaptation of WHODAS 2.0

The cross-cultural adaptation of the Nepali version of WHODAS 2.0 involved a stepwise translation of the original English version into Nepali using a standard forward–backward translation protocol [38], and subsequently, a cultural validation procedure [5]. Approval for translation and reproduction of the WHODAS was received from the WHO (ID 307,778, date: 18 December 2019) [39]. The entire process is outlined below:

#### Forward translation

Two native translators with good knowledge of English translated independently the original version of WHODAS 2.0 into Nepali.

#### Synthesis

Any discrepancies were discussed between the two translators. Items in need of modifications in the cultural adaptation were considered and resulted in a synthesized single Nepali version.

#### Back translation

Two other bilingual translators translated the synthesized Nepali version independently back into English.

#### Expert committee meeting

All four translators and the researchers met to discuss the appropriateness of the translation to ensure equivalence to the original English version of WHODAS 2.0. By consensus, the meeting resulted in a pre-final Nepali version of WHODAS 2.0.

#### Pre-testing

The pre-final Nepali version was tested on 30 consenting volunteers visiting the Internal Medicine OPD of DH (13 males, 17 females; 12 with psychiatric disability, 10 with chronic physical illnesses, 8 without any such illnesses) to evaluate the comprehensibility of the pre-final scale. To ascertain clarity in the item-formulations, they were asked about the meaning of each item and about any difficulties they encountered.

Further reconciliation of the translated materials and language amendments resulted in a quality assured culturally adapted Nepali WHODAS 2.0 instrument that ultimately was used for the validation in the present study.

### Data analysis

The data were analysed using the IBM SPSS Statistics 21, Chicago, USA.

The Kaiser–Mayer–Olkin’s (KMO) measure of sampling adequacy was used to assess the suitability of the data for factor analysis; a value > 0.6 is considered sufficient for factorability of the correlation matrix [40]. Bartlett’s test of sphericity was used to ensure that the correlation matrix would not contain problematic relationships between items.

By using factor analysis with varimax rotation, we checked the number of factors and the distribution of Eigenvalues of the items. To avoid factor oversampling, we visually inspected the scree plot [41] and carried out a parallel analysis [42].

Construct validity was sought by exploratory factor analysis in order to obtain the one-factor solution for the WHODAS 2.0 and by having all items correlate > 0.3 to the latent factor.

Content validity implies that the items are representative of the entire feature that the test aims to measure. This was tested by the item-to-sum correlations and by checking that no major inconsistencies were present in the scale-items of the main factor.

Criterion validity was explored by correlating WHODAS 2.0 with another variable that covered related features. We decided to use the quality of life instrument (WHOQoL-8) with its total score and four domains scores to assess the concurrent criterion validity. Cronbach alpha was used to evaluate internal consistency of the scale. An alpha ≥ 0.70 was set as the threshold for satisfactory reliability [43].

We used simple addition of the 12 WHODAS 2.0 item scores to obtain the total disability score [8]; Kolmogorov–Smirnov test did not show a normal distribution of the disability score. Accordingly, we used Mann–Whitney U test to check whether gender significantly influenced the distribution of the disabled vs. non-disabled and also to check the difference between physical vs. psychiatric disability with regard to the WHODAS 2.0 total scores.

The p-value < 0.05 was considered to indicate statistical significance in all computations.

## Results

### Cross-cultural translation and adaptation of WHODAS 2.0

The “Instructions to the participants” were translated into Nepali and used to facilitate communication and develop rapport with the interviewees. The expert committee aimed for simplicity in the item translation. Persistently, they used common spoken language. Semantic issues arose in three items. To arrive at analogous expressions in Nepali, the translators exchanged “taking care” with “**carrying out**” household responsibilities in S2, “washing” replaced with “**bathing**” in S8, and “dealing with” was replaced by “**being accustomed to**” strangers in S10. The expressions in bold were found to be the more suitable in the Nepali context. Only “**work”** from the expressions “work/school” in S12 was retained unchanged in the final Nepali version; the age inclusion criterion was 18 years and above, and at that age most would leave secondary school and join college.

### Participants

In total, 149 persons [mean age: 40.6 (12.8); 43.6% males, 56.4% females] consented to participate in the study. Almost four-fifths were married (79.9%); none of the participants were divorced or widowed. More than three-fourths were from the cities (77.2%); 65.8% were literate. Almost three-fifths were living in a nuclear family (57%), average family size was 5.2 (2.5) persons. Two-fifth of the participants belonged to the lower SES (40.3%). Out of the total, 38.3% accompanied a patient when coming to the Psychiatry or Medicine OPD, and they had no disability. More than three-fifths of the total was having a disability; 36.2% had psychiatric disorders, 20.8% had a physical illness, and 4.7% had both kinds of disorders (Table 2).

**Table 2:** Socio-demographic and disability information about Nepali participants (N = 149)

Variables	Number (%)
Age (in years)*	
Below 40 years	75 (50.3)
40 years and above	74 (49.7)
Sex	
Male	65 (43.6)
Female	84 (56.4)
Marital status	
Unmarried	30 (20.1)
Married	119 (79.9)
Literacy status	
No (Illiterate)	51 (34.2)
Yes (Literate)	98 (65.8)
Residence	
Rural	34 (22.8)
Urban	115 (77.2)
Family type**	
Nuclear	85 (57)
Joint or extended	64 (43)
Occupational status	
Employed	68 (45.6)
Housewife (Homemaker)	56 (37.6)
Unemployed	25 (16.8)
Socio-economic status***	
Lower	60 (40.3)
Middle	44 (29.5)
Upper	45 (30.2)
Disability related factors	
No disability	57 (38.3)
Psychiatric disorders only	54 (36.2)
Physical disorders only	31 (20.8)
Both psychiatric and physical disorders	7 (4.7)

*Mean (SD): 40.6 (12.8); **Average family size: 5.2 (2.5); ***Mean Kuppuswamy score: 12.9 (6.0)

KMO’s measure of sampling adequacy was 0.84 and Barlett’s test of sphericity was significant (chi-square = 487.91, df = 66, *p* < 0.001) indicating suitability of the data for factor analysis.

### Construct validity

At first, exploratory factor analysis with varimax rotation resulted in a three-factor solution with eigenvalues > 1. The first extracted factor (F1) had higher correlations for four items (1, 7, 8, and 9); they were identifying problems associated with basic daily physical functions: problems related to standing, walking, dressing, washing etc. The second extracted factor (F2) showed higher correlations on five items (2, 3, 4, 6, and 12) related to the person’s functions in a wider context: participating in household activities, learning new tasks, joining community functions, tasks requiring concentration and day-to-day tasks. The third extracted factor (F3) was related to the person’s social capacity; they had higher correlations on items 10 and 11 only. Item 5 dealt with emotional aspect of health; it had a relatively higher correlation with F1. The cumulated explained variances of three factors were 28.6%, 51.8%, and 65.3% (Table 3).

**Table 3:** Item-to-factor correlations from free and forced rotated factor analyses of WHO DAS 2.0 (12-items version) translated into Nepali within those with a disability (n = 92)

Items	Text of the items	Rotated three-factors solution*			Forced one-factor solution**
		Factor 1	Factor 2	Factor 3	
	In the last 30 days, how much difficulty did you have in:				
1	Standing for long periods such as 30 min?	0.77	0.29	0.04	0.75
2	Taking care of your household responsibilities?	0.46	0.66	0.10	0.78
3	Learning a new task, for example, learning how to get to a new place?	0.09	0.81	0.03	0.58
4	How much of a problem did you have in joining in community activities (for example, festivities, religious or other activities) in the same way as anyone else can?	0.17	0.69	0.12	0.59
5	How much have you been emotionally affected by your health problems?	0.55	0.37	0.12	0.66
6	Concentrating on doing something for ten minutes?	0.55	0.57	0.16	0.79
7	Walking a long distance such as a kilometer [or equivalent]?	0.73	0.37	0.23	0.82
8	Washing your whole body?	0.72	0.39	0.03	0.77
9	Getting dressed?	0.87	− 0.06	0.11	0.63
10	Dealing with people you do not know?	0.08	0.01	0.88	0.31
11	Maintaining a friendship?	0.15	0.25	0.81	0.49
12	Your day-to-day work/school?	0.40	0.58	0.25	0.72
Explained variance % (cumulative percentage for the three-factor solution)	28.6	51.8	65.3	45.4	
Cronbach alpha	0.89				

^*^Principal component analysis with Varimax rotation; **Principal component analysis

Factor analysis has been criticized for oversampling factors. Accordingly, we made a more detailed exploration of the factor structure [41, 44]. Visual inspection of the scree plot indicated one major factor of the Nepali-translated WHODAS 2.0 12-item version. This one-factor solution was confirmed by the computations of a parallel analysis [42]; only the first eigenvalue in the exploratory factor analysis was greater than the average eigenvalues in the primary analysis column. Hence, parallel analysis indicated only one factor.

Based on the indication from the parallel analysis, we made a forced one-factor analysis. As displayed in the Table 3, the items had higher correlation values (nearer to or above 0.58) except items 10 (0.31) and 11 (0.49). The explained variance of the one-dimensional version was 45.4%. The one-dimensional construct of the WHODAS 2.0 was validated by the parallel analysis in combination with the emerging correlations of the forced one-factor solution.

### Content and criterion validity

As seen in Table 4A, content validity was demonstrated by an adequate level of inter-correlations between the six domains of the WHODAS2.0. Moreover, all domains correlated well with the WHODAS 2.0 total score.

**Table 4:** Content (A) and Criterion (B) validity of the Nepali-version WHODAS 2.0 instrument (N = 149)

WHODAS 2.0 Domains	A. Correlation matrix of WHODAS 2.0 domains							B. Correlations between WHODAS 2.0 and WHOQoL-8 domains				
	WHODAS 2.0 Domains							WHOQoL_8 Domains				WHOQoL-8 Total score
	Do1	Do2	Do3	Do4	Do5	Do6	Total score	Global	Physical	Psychosocial	Environmental	
Do1. Cognition	1	0.651^a^	0.551^a^	0.382^a^	0.593^a^	0.691^a^	0.839^a^	− 0.274^a^	− 0.241^b^	− 0.263^c^	− 0.358^a^	− 0.375^a^
Do2. Mobility		1	0.613^a^	0.323^a^	0.599^a^	0.682^a^	0.854^a^	− 0.319^a^	− 0.220^b^	− 0.336^a^	− 0.360^a^	− 0.397^a^
Do3. Self-care			1	0.234^c^	0.513^a^	0.557^a^	0.739^a^	− 0.352^a^	− 0.220^b^	− 0.288^a^	− 0.281^c^	− 0.372^a^
Do4. Getting along				1	0.314^a^	0.377^a^	0.548^a^	− 0.094	− 0.001	− 0.173*	− 0.090	− 0.097
Do5. Life activities					1	0.623^a^	0.789^a^	− 0.367^a^	− 0.236^c^	− 0.253^c^	− 0.331^a^	− 0.387^a^
Do6. Social participation						1	0.854^a^	− 0.371^a^	− 0.180^c^	− 0.335^a^	− 0.410^a^	− 0.399^a^
WHODAS 2.0 Total score							1	− 0.383^a^	− 0.240^c^	− 0.355^a^	− 0.399^a^	− 0.441^a^

Spearman’s correlation coefficient; ^a^*p* < 0.001, ^b^*p* < 0.01, ^c^*p* < 0.05

All four domains of the WHOQoL-8 showed significant inverse correlations with the six domains of the WHODAS 2.0 item, except the “getting along” domain. The total scores of WHODAS and WHOQoL-8 were also inversely correlated (ρ = −0.441; *p* < 0.001) (Table 4B).

### Disability scores

#### Gender differences

The mean disability score of the male and female population were 9.5 (SD 7.9) and 15.7 (SD 9.9) respectively. The respective median scores were 8 (range 4–13.5) and 14 (range 8–24). The gender difference was significant with regard to the total WHODAS score (U = 1698.5; *p* < 0.001) and significant for the six domain-specific scores as well.

#### Differences between disabled and non-disabled

The mean disability scores among the disabled and non-disabled population were 14.5 (SD 9.9) and 10.6 (SD 8.7) respectively. The respective median scores were 12 (range 6–22) and 8 (range 4–16). A significant difference (U = 2002.5; p = 0.015) was found between the total scores of those with a disability and those without. Significant differences in the WHODAS domain scores between the disabled and non-disabled was found for cognition, self-care, getting along, and life activities.

#### Differences between psychiatric and physical disability

The mean disability scores among the psychiatric and physical disabled population were 13.2 (SD 9.7) and 16.7 (SD 10.4) respectively. The respective median scores were 10 (range 6–19.2) and 14 (range 8–27). However, those differences were not statistically significant. A significant difference in the mobility domain was seen (U = 539.5; p = 0.006), which mainly covers activities requiring physical capacities: standing and walking.

### Reliability

As shown in the Table 3, the Cronbach alpha value was 0.89 for the 12-items version.

## Discussion

After the principal component analysis, visual inspection of the scree plot and parallel analysis, only one factor was found to be viable for the culturally adapted Nepali-version of WHODAS 2.0. In the one-factor solution, all 12 items of the Nepali-version WHODAS 2.0 had adequate correlations. A one-factor solution is in line with both the original English version [1, 8] and with the majority of the cross-cultural applications developed for this instrument [3–5, 11–13] and underscores the construct validity. In addition, the psychometric qualities of the Nepali one-dimension structure of the scale show adequate reliability, content validity, and criterion validity. The one-factor solution in the Nepali-version of the WHODAS 2.0 will allow international comparisons. No significant difference was found between physical and psychiatric disability. This finding underscores the generalized disability concept measured by the scale; it encompasses both physical and psychiatric disability.

In the Nepali version, three items required culturally adapted alterations in the idiomatic statements; they were similar to the Hebrew-version [4]. The culturally adapted version was readily accepted by the general Nepali population. A recent international review on the WHODAS 2.0 argued that some items may show correlations different from rest due to cultural sensitivity and individual bias inherent with the subjective reporting procedure [3]. Likewise, the Australian study underlined the possibility of cross-national variations in the standards for disability measures [9]. Even within-country variations due to word syntax, communication patterns, and differences in terminology may affect the instrument adaptation [11]. In the same vein, socio-cultural issues typical for Nepal may have affected the interview process [30], eventually resulting in the relatively lower correlations of the two items related to social attributes (items 10 and 11).

The Cronbach alpha of the original WHODAS 2.0 was 0.86 [8]. Validity studies in other parts of the world also found high alpha values such as 0.89–0.98 in China [11], 0.83–0.92 in Sweden [10], 0.84–0.93 in other European countries [16], 0.9 in an international review [3], and 0.90–0.97 in the cross-cultural study from LAMI countries [13]. The internal consistency reliability of the Nepali-version of WHODAS 2.0 (0.89) was acceptable [43] and in line with findings in the aforementioned studies.

In keeping with most validity studies of people with either mental disorders [9, 16] or chronic physical conditions [18, 21–23] or both [14], the Nepali WHODAS 2.0 version preserved the validity of the general disability criterion. The Nepali scores were higher among persons with any disability, physical, psychiatric, or both, in comparison to those without any disability. However, the Nepali-version did not discriminate between the physical and psychiatric disability. The Australian study identified higher disability scores among those with mental disorders than among those with physical disorders [9]. A study from France showed almost similar scores between the physical disorders and the psychiatric ones [19].

Significant correlations of the WHODAS domains with the total disability score indicate content validity of the Nepali instrument. This was in keeping with the findings from a Chinese study [45]. In addition, inverse correlations with the QoL score demonstrate criterion validity in the Nepali version; the disabled have lower quality of life. Similar findings are seen in other cross-cultural studies [3, 11, 45].

Our study has certain methodological shortcomings. Being a hospital-based study with a convenience sample, the findings may perhaps not be generalizable to the general community. Convenience sampling of accompanying persons as controls can imply a certain recruitment bias. The family dynamic of chronically disabled persons may have affected family members to some degree, including the accompanying person. To minimize this potential bias, we tried to include consecutive accompanying person who did not have a past or family history of mental illnesses. However, if affected by the family dynamic, the accompanying persons would score closer to the disabled participants on the test which would tend to blur the differences between the two groups.

Although the WHODAS 2.0 can be self-administrated, we used the interview method. This is unavoidable in view of the literacy status of many participants. Earlier studies elsewhere have also modified the administration of the WHODAS 2.0 in a variety of ways; i.e., online or web-based approach were used in Sweden [10], while online or a mailed questionnaires were used in China [11]. Due to the robustness of the scale, we tend to believe that our interview approach has not essentially affected the outcome. In the past, we have adopted similar data collection procedures in other studies with good outcome [46, 47].

As we focused on the one-factor solution of the WHODAS instrument, we did not carry out a confirmatory factor analysis. We did not attempt to obtain a test–retest reliability. A certain time would have to pass before retesting would make sense. If the time window was too narrow, the chance is that the participants could complete the inventory from their memory. Due to lack of resources, test–retest would have to depend on self-administration by mail. With the low literacy rate, this would not be a viable solution. Accordingly, we explored the concurrent criterion validation using the QoL instrument which already was a part of our survey battery. There are other, well-established disability inventories that could have been used, but they do not yet exist in Nepali.

This is a pioneering attempt to develop a culturally suitable and valid instrument for measuring disability in Nepal. It followed standard procedures in the translation and applied well-established statistical psychometric procedures for assessing the reliability and validity. The study has demonstrated enough ground to recommend use of the WHODAS2.0 in the future, and this study expands the pool of translated, culturally adapted and validated tools in the country.

## Conclusions

The Nepali-version of WHODAS 2.0 has been found to be a psychometrically sound, reliable, and valid instrument that can be used to assess general disability in the Nepali speaking population. The instrument has been culturally adapted for use in both epidemiological research and clinical settings.

## Data Availability

Personal identification details of the participants were separated from the completed questionnaires and stored in a locked room at the Kathmandu University School of Medical Sciences (KUSMS). No information relating to identifiable individuals was disseminated beyond the few researchers immediately involved in the study.

## Abbreviations

DH: Dhulikhel Hospital
ICD: International Classification of Diseases
ICF: International Classification of Functioning, Disability and Health
IRC-KUSMS: Institutional Review Committee of Kathmandu University School of Medical Sciences
KMO: Kaiser–Mayer–Olkin
LAMI: Low-and-middle-income
OPD: Out-patients Department
QoL: Quality of life
SES: Socio-economic status
WHO: World Health Organization
WHODAS: World Health Organization Disability Assessment Schedule
WHOQoL-8: World Health Organization Quality-of-Life 8-question scale
WMH: World Mental Health
